# Features of the Macro-, Micro-, and Fine Structure of the Nickel Superalloy Product Material Formed by the Method of Electron Beam Additive Manufacturing

**DOI:** 10.3390/ma15248882

**Published:** 2022-12-12

**Authors:** Sergey Fortuna, Denis Gurianov, Sergey Nikonov, Konstantin Ivanov, Yury Mironov, Andrey Vorontsov

**Affiliations:** Institute of Strength Physics and Materials Science, Siberian Branch of the Russian Academy of Sciences, Pr. Akademicheskii, 2/4, 634021 Tomsk, Russia

**Keywords:** superalloy, microstructure, nanoscale precipitations, electron beam technology, additive manufacturing

## Abstract

In the present work, the products in the form of vertical walls were made of heat-resistant nickel-based superalloy ZhS32 via the method of electron beam additive technology. Unidirectional printing strategy was applied. The effect of heat input and 3D printing strategy on the macrostructure, dimensions, and morphology of microstructure elements was established. It was shown that the additive product material has a directed macrostructure. The only exclusion was the final layer with a thickness of no more than 3.5 mm. The directed macrostructure consisted of dendrites oriented predominantly along the crystallographic direction {001} of the primary dendrite arms. The misorientation of the dendrite axes did not exceed 9 degrees. The angle between the predominant dendrite growth direction and the normal to the substrate was 23 degrees. The average primary dendrite arms’ spacing increased monotonically from 16 µm at 5 mm from the substrate to 23 µm in the final layers of the product material (the overall height was 41 mm). It was found that the average size of γ’ (Ni_3_Al)-phase precipitations in the form of nanoscale and submicrocrystalline cuboids varied in the range of 76 to 163 nm depending on the distance from the substrate. The size of γ’-phase precipitations reached a maximum at about 30 mm from the substrate, while in the final layers of the product material, the average cuboid size did not exceed 135 nm. Extreme dependence of the size of γ’-phase precipitations on the height of the product followed from a combination of a given monotonic decrease in heat input and heat accumulation in the product material as it formed, as did additional heat removal by means of radiation during formation of the final layer of the product without re-melting. Chemical elements of the austenitic steel substrate material were not detected in the product material more than 8 mm from the substrate. There were no macrodefects, such as voids, in the entire volume of the product material.

## 1. Introduction

Nickel-based heat resistant superalloys are in demand in many industrial applications for which creep resistance at high temperatures and in aggressive environments is required. Due to the high cost of such alloys, additive technologies are a more attractive method of manufacturing products operated in high-temperature conditions than traditional methods, such as investment casting, hot forging, and complex machining [1,2].

It is known [1,3] that due to the high temperature gradient, a textured structure is formed during additive growth. During solidification, grains/dendrites are formed that grow along the <100> crystallographic direction with an inclination towards the thermal source movement at a certain angle [1], which leads to anisotropy not only of mechanical properties but also of corrosion properties [2]. According to [4], in the nickel-based superalloy Hastelloy X, the highest strength and yield strength limits are achieved along the <111> crystallographic direction and the relative elongation along <110>. In some cases (for example, in the production of gas turbine engine blades), the anisotropy of properties is necessary because the resistance to creep and thermal fatigue is higher in products with a directional or monocrystalline structure [4]. It is worth noting that the crystallization conditions in additive formation allow the removal of grain selectors used in casting.

The heat-resistant properties of nickel-based superalloys are determined by an ordered γ’-phase (intermetallic compound AlNi_3_ of the binary Al-Ni system) with an L1_2_ type superstructure based on the FCC crystal lattice (symmetry group Pm3̅m). The γ’-phase precipitates prevent the movement of dislocations during plastic deformation, thereby strengthening the material [5]. This phase can vary in size, morphology, and chemical composition, which in turn will affect the properties of the product. The morphology of γ’-phase precipitations varies in a wide range from spherical to cubic and, depending on the chemical composition and operating conditions, can also take the form of raft structure after prolonged stress at elevated temperatures [6].

Currently, many different approaches to additive manufacturing have emerged. A considerable amount of modern research is devoted to this direction, on the basis of which the following remaining challenges of additive technologies can be noted:

- Presence of macrodefects, such as voids;
- Ensuring a given microstructure and phase composition of the product material;
- Ensuring a given chemical composition of the product material;
- Ensuring a given geometry of the product;
- Ensuring a given waviness and roughness of the external and internal surfaces of the product.

Of these five problems, the first three cannot be corrected by any subsequent technological operations except partial correction of the microstructure and phase composition.

At the current stage of technological development, the last two problems can be corrected by means of finishing machining. The first two problems can only be solved by a reasonable combination of technological parameters in 3D printing. It is not possible to correct the microstructure or/and chemical composition in the finished product. In the additive process, products made of superalloys (nickel-based heat-resistant alloys) can be susceptible to cracking under conditions of rapid cooling [7,8,9]. Earlier studies [10,11] made it possible to determine the ranges of heat input values for the formation of additive products that do not contain macrodefects and have satisfactory compliance with the given product geometry.

For gas turbine engine blades, a directed—or better, monocrystalline—material structure of nickel-based superalloy products is considered justified. Both directional solidification casting technologies and additive technologies use special techniques to provide directional or monocrystalline structures for the blade material. In casting technology, this is a very slow movement of the solidification front in the molds. In the case of additive technologies, it is a set of related technological parameters, such as printing strategy (determined by printing direction relative to the substrate and energy source); the value of the flow energy (determined by the ratio of beam current values, accelerating voltage, and speed of working table movement relative to the energy source); filament feeding speed; and direction and value of temperature gradient (affected by conditions of heat removal from the melt bath).

The quality criteria of the world manufacturers of aircraft engines require that the value of the misorientation angle depending on the application be below 15, but better less than 8 degrees in order to recognize the blade as suitable for use in an engine [12,13].

In order to establish the possibility of forming an additive product of heat-resistant nickel-based superalloy ZhS32 (analog of Rene 150 superalloy) with a directed structure, the present study was carried out. In the course of the work the presence of macro-defects, elemental composition, and the features of macro-, micro-, and fine structure of the additive product material were determined.

## 2. Materials and Methods

The product was formed at the wire-feed electron beam additive setup developed at the Institute of Strength Physics and Materials Science SB RAS [14]. From the initial material in the form of a large-sized ingot of nickel-based ZhS32 heat-resistant alloy (chemical composition is presented in Table 1) by means of wire electric erosion cutting, bars of square cross-section with size 3.0 × 3.0 mm^2^ and length about 146 mm were obtained. After the removal of erosion products, the cleaned rods were loaded into a special feeder of an electron beam machine [15]. By feeding the rods into the focus of the electron beam and moving the working table, additive wall products were formed layer-by-layer on a substrate of SS321 austenitic steel (chemical composition is presented in Table 2).

**Figure 1:**
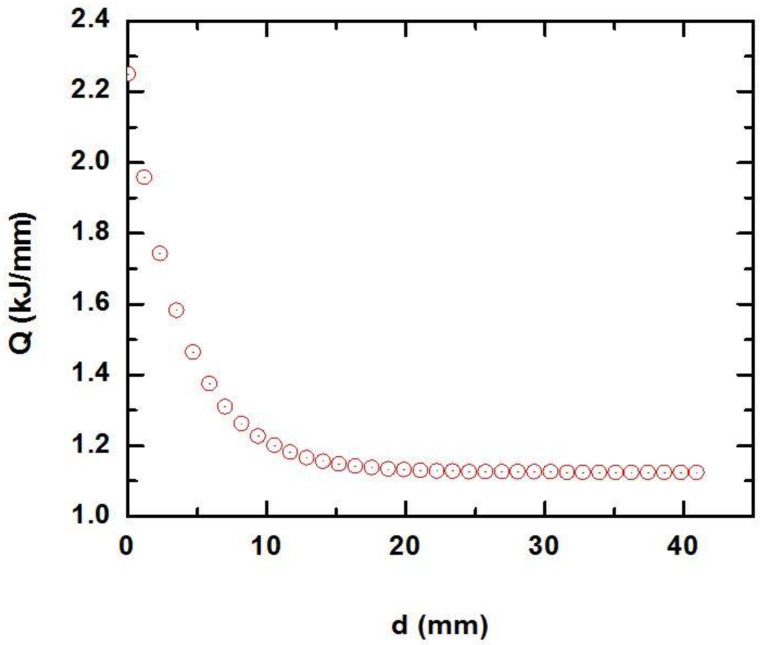
Dependence of the heat input values on the distance to the substrate.

During additive product formation (3D printing), the electron beam current varied from 25 to 13 mA. At the same time, the accelerating voltage of the electron beam and the speed of movement of the working table remained unchanged during the entire process of 3D printing and were 30 kV and 20 mm/min, respectively. The combination of the indicated values of technological parameters resulted in the present value of the thermal absorption [17] of the electron beam in the range from 2.25 to 1.13 kJ/mm. The dependence of the calculated values of heat input on the distance to the substrate in the process of additive formation of the product from ZhS32 superalloy is shown in Figure 1. Calculation of the heat input values was carried out according to the equation [17]:

(1) $$Q=\frac{60\times U\times I}{1000\times V}$$

where *U*—accelerating voltage, kV; *I*—electron beam current, mA; *V*—working table speed movement, mm/min.

The strategy of unidirectional 3D printing was followed (each subsequent layer was deposited in the same direction as the previous one during the product formation).

**Figure 2:**
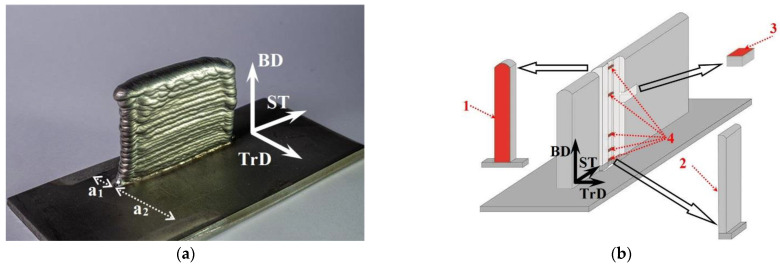
General view of products obtained by wire-feed electron beam additive manufacturing (**a**) and schematic view of specimens cut out for structural studies (**b**). Metallographic studies were carried out on prepared surface 1. Metallographic and SEM studies were carried out on prepared surface 2. X-ray studies were conducted on surface 3. TEM and SEM studies were conducted on surface 4. BD—building direction, ST—scanning trajectory, TrD—transverse direction.

A simplified additive product in the form of a vertical wall was formed in 36 unidirectional passes, and its largest dimensions were 56.0 × 40.9 × 11.1 mm^3^ (length–height–width) (Figure 2a).

Templates (blanks) were cut from the formed additive product for subsequent production of longitudinal and transverse (parallel and perpendicular to the 3D printing trajectory) thin sections for structural studies using light and scanning electron microscopy. Metallographic sections were prepared via mechanical grinding and polishing, with chemical etching with Marble reagent (50 mL HCl + 10 g CuSO_4_ + 50 mL H_2_O) at the final stage of preparation. The macro- and microstructures of the additive material were studied using an AXIOVERT-200MAT light microscope (Zeiss, Jena, Germany), an LEO EVO 50 (Zeiss, Germany), and an Apreo S LoVac with an EDS + EBSD analytical system (Thermo Fisher Scientific, Waltham, MA, USA). The fine structure and phase composition were studied using a JEM-2100 transmission electron microscope (JEOL Ltd., Tokyo, Japan). Objects for electron microscopic studies in the form of thin foils were prepared via the ionic thinning method on the EM-09100IS machine (JEOL Ltd., Japan). In addition, structural investigations were performed using a DRON-7 X-ray diffractometer (Burevestnik, St. Petersburg, Russia).

The elemental composition of the material in the initial state was determined using a Niton XL3t GOLDD++ X-ray fluorescence spectrometer (Thermo Fisher Scientific, USA). The elemental composition of the additive product material was determined in areas at different distances from the substrate during SEM and TEM studies using INCA analyzers (Oxfords Instruments, Abingdon on Thames, UK, GB).

The orientation of crystal lattice γ- and γ’-phases (as the main phase components of the additive material of ZhS32 superalloy) relative to the growth direction of the additive product was determined via X-ray diffractometry in CoKα-radiation (1.78897 Å) at asymmetric imaging geometry [18]. For this purpose, the intensities of reflections from the {002} γ- and γ’-phase plane family were recorded when the orientation of the sample changed (rocking angle [13]). The intensities were used to determine the angular deviation of the crystallographic direction of <001> γ- and γ’-phases relative to the normal to the substrate plane, coinciding with the additive product growing direction (denoted as BD in Figure 2 and below) along the 3D printing path (denoted as ST—scanning trajectory in Figure 2 and below). X-ray examination was performed on a specially prepared polished metallographic sample (surface 3 in Figure 2b). It should be noted that surface 3 was perpendicular to the BD and was located at a distance of 29.2 mm from the substrate, outside the influence zone of the substrate material (as shown below).

It should be noted that in addition to the above characteristic directions BD and ST, there are two more designations: TrD—transverse direction, i.e., the direction transverse to the ST, and GD—dendrite growth directions.

## 3. Results and Discussion

### 3.1. Features of the Macro- and Microstructures

Visual examination of the additive product formed using electron beam additive technology methods did not reveal any cracks on the outer surfaces of the product (Figure 2a). Note that detailed examination by means of light and scanning electron microscopy confirmed the absence in the product material of such defects as discontinuities (cracks, pores, non-melting, inclusions, etc.).

**Figure 3:**
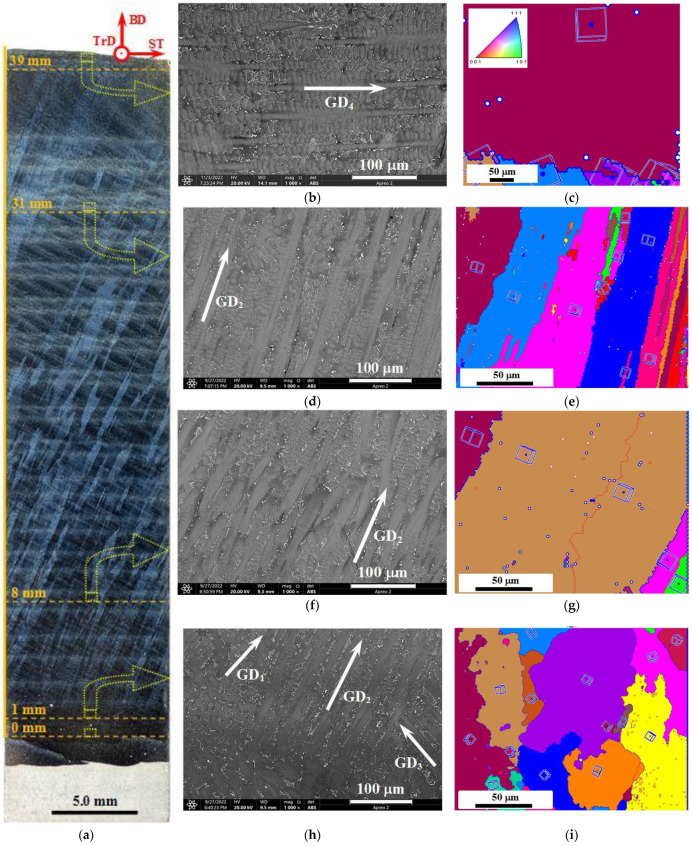
Macro- (**a**), microstructure (**b**,**d**,**f**,**h**), and EBSD maps (**c**,**e**,**g**,**i**) of material of products obtained via wire-feed electron beam additive manufacturing at a distance from the substrate of: 39 mm (in the final layer)—(**b**,**c**); 31 mm—(**d**,**e**); 8 mm—(**f**,**g**); 1 mm (in the transition layer)—(**h**,**i**). BD—building direction, ST—scanning trajectory, TrD—transverse direction, GDi—direction of dendrite growth. The closed dashed lines show the localization of the areas subjected to detailed studies by means of SEM and TEM.

In a cross section perpendicular to the TrD direction (Figure 3a), the macrostructure of the product material in the form of unidirectional dendrite colonies is clearly visualized. The dendrite colonies extend from the transition layer at the substrate to the final (penultimate and last) layers of the product and slope in the direction of the 3D printing ST (Figure 3a,d,e–g). According to the results of measurements on macroscopic images, the calculated value of the average angle of inclination of dendrite colonies relative to the vertical direction of growing the product BD was 23 ± 3 degrees. Earlier studies [19] on the example of ZhS6U superalloy showed that the slope of dendrite colonies in the additive product material is caused by the curvature of the solidification front in the melt bath and is determined by the filament feed rate and the working table movement speed in the process of 3D printing.

In the transition layer, a mainly multidirectional structure was formed (Figure 3h,i); moreover, the influence of dissolved chemical elements of the substrate is manifested here, which will be shown below. The pre-final additive layer also reveals a mixed structure of multidirectional dendrite colonies, and in the final additive layer, the dendritic colonies are directed predominantly horizontally (codirected with the ST direction), see Figure 3b,c. The directional growth of the dendrites is due to the epitaxial crystallization mechanism. In the material structure of the additive product, the dendritic colonies have only azimuthal disorientations relative to each other and, consequently, relative to the dendrite growth directions (Figure 3e,g). It is the presence of azimuthal disorientations that provides a characteristic contrast on metallographic thin sections and allows visualization of the dendritic colonies (Figure 3a). At the same time, each of the dendritic colonies is formed by dendrites in direct contact with each other and with small-angle (less than 15 degrees) azimuthal and/or radial disorientations relative to each other (Figure 3c,e,g,i). In Figure 3, low-angle boundaries are shown with thin red lines and high-angle (>15 degrees) boundaries with blue lines.

Light and scanning electron microscopy reveal the internal microstructure of dendrite colonies. As can be seen from the SEM images presented in Figure 3b,d,f,h, the microstructure of dendrite colonies is formed by the primary dendrite arms parallel to each other and perpendicular to them secondary arms. The primary arms extend to the full length of the colonies, and the secondary arms fill the space between them.

**Figure 4:**
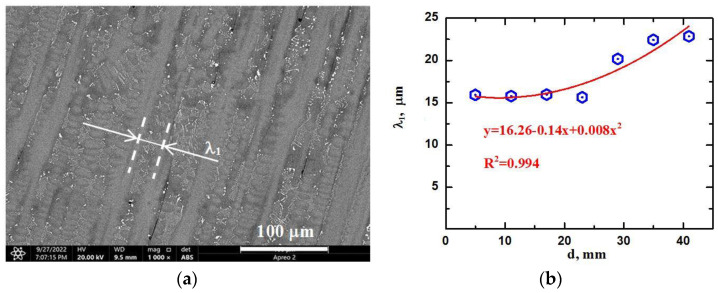
Characteristic SEM image of the dendritic structure of the additive ZhS32 superalloy material (**a**) and the dependence of the average primary dendrite arm spacing λ_1_ on the distance to the substrate in the product material (**b**).

Note that at distances of 5 to 38 mm to the substrate, the macrostructure and microstructure of the product material appear to change monotonically in terms of an increase in the size of structural elements in the form of dendrite colonies and dendrites composing them. Measurements of primary dendrite arm spacing showed their increase from 16 to 23 μm at distances of 5 and 40 mm to the substrate, respectively. The dependence of the average primary dendrite arm spacing λ_1_ on the distance to the substrate is shown in Figure 4. Mathematical processing of the experimental results of the measurements of λ_1_ allowed it to establish an approximation by a second-order polynomial (which differs little from linear), with the coefficient of determination R^2^ = 0.994.

**Figure 5:**
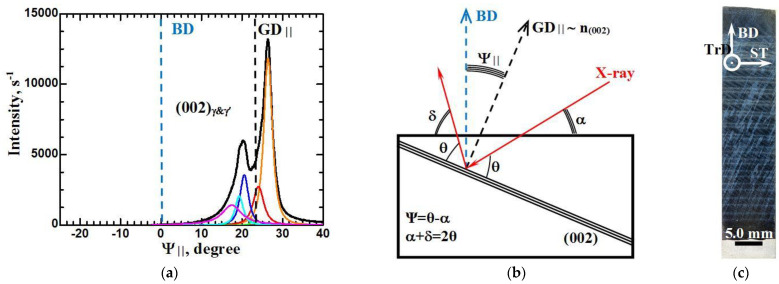
Distribution of the intensity of X-ray radiation diffracted from the crystallographic planes (002) γ- and γ’-phase as a function of the rocking angle relative to the BD (**a**), X-ray imaging scheme (**b**), and image of the macrostructure (**c**) in a section perpendicular to the TrD.

**Figure 6:**
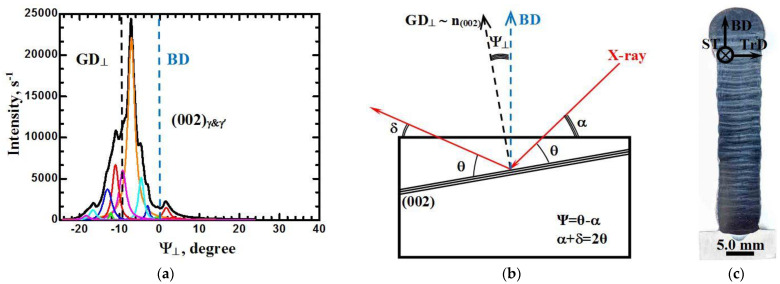
Distribution of the intensity of X-ray radiation diffracted from the crystallographic planes (002) γ- and γ’-phase as a function of the rocking angle relative to the BD (**a**), X-ray imaging scheme (**b**), and image of the macrostructure (**c**) in a section perpendicular to the ST.

Another notable feature of the material macrostructure of additive product is the following. The longitudinal and, especially, transverse metallographic sections (Figure 5c and Figure 6c, respectively) clearly show light bands parallel to the substrate in the macrostructure of the product material. The stripes decorate the layers of the printed product. At the same time, the thickness of the layers is of an alternating nature. The nature of such structural features has been established in recent work [20]. Here, it will be noted that such contrast in the macrostructure images is due to the fact that half a bar of the raw material is required to form one layer of the additive product. Before the formation of each odd layer (except for the first one), there is a technological operation of dumping the cinder of the consumed rod and supplying a new rod. This leads to an increase in the time interval between the end of the formation of the previous layer and the beginning of the next one by about five times, from 3 to 16 s. Increasing the delay results in a greater reduction in material temperature of the last formed layer, and material with a lower temperature is re-melted to a shallower depth, resulting in an increase in their thickness.

From the evaluation of the above features of the macro- and microstructures of the material of the additive product under study, the following conclusion can be drawn. The material of the additive superalloy product has mostly unidirectional structure in most of its volume, except for the transition layer at the substrate, which is about 1.5 mm thick, and the final layers have a thickness not exceeding 3.5 mm. At the same time, ensuring precisely directed—or better, monocrystalline—material structure of products made of nickel superalloys is an extremely important and urgent goal for scientific teams and leading industrial enterprises [21]. It should be noted that in practical application, it will be necessary to remove the initial layers at the substrate affected by the substrate elements as well as the final layers of the product with different macro- and microstructures.

Quantitative values of the deviation of the GD direction from the additive BD, as well as the disorientation of the dendrite colonies relative to the GD, were determined by means of X-ray diffractometry using the rocking method, as described in Section 2, in two planes: in the plane perpendicular to the TrD direction and in the plane perpendicular to the ST (Figure 5 and Figure 6, respectively). In these figures, the angular deviations of the GD relative to the additive BD in these planes are shown as Ψ_∥_ and Ψ_⊥_, respectively.

The results presented in Figure 5 and Figure 6 can be supplemented with the following comments.

In the section perpendicular to TrD, the direction of the preferential growth of dendrites is deviated relative to the vertical direction of growing the additive product. The X-ray orientation reflex from the family of (002) γ- and γ’-phase planes has a complex morphology and is a composition of six reflexes. The gravity center of the composite orientation reflex falls at the value of 23 degrees (which coincides with the results of measurements on macroscopic images). That is, the preferential direction of dendrite growth in this GD plane is tilted from the vertical direction BD by the specified angle in the direction of unidirectional 3D printing (along the ST direction).

The disorientation of the crystals (dendrite colonies) relative to the center of gravity of the compound reflex (coinciding with the GD direction) does not exceed 9 degrees. No orientational boundaries are detected, i.e., the boundaries between these dendritic colonies are low-angle. The volume fraction of dendrite colonies forming the peak with the highest intensity is about 0.54. This peak is deflected relative to the GD direction in the ST direction by 3.4 degrees. Another group of dendrite colonies with a volume fraction of about 0.12 is deviated in the same direction by 1 degree. Four groups of dendrite colonies with a total volume fraction of about 0.34 are deflected antiparallel to the ST direction at angles from 0.5 to 5.5 degrees.

In the section perpendicular to ST, the direction of the preferential growth of the dendrites is also deviated from the vertical direction of the additive growth. The orientation reflex from the {002} γ-and γ’-phase plane family has a complex morphology and is a composition of twelve detectable orientation reflexes. The center of gravity of the composite orientation reflex falls at a value of negative 9 degrees, i.e., the preferential direction of dendrite growth in this plane GD is tilted from the vertical BD by the specified angle antiparallel to the TrD.

The misorientation of the crystals (dendritic colonies) relative to the gravity center of the composite orientation reflex (coinciding with the GD), taking into account low-intensity reflexes, is about 22 degrees. As in the previous case, the boundaries between dendrite colonies are low-angle. The volume fraction of dendrite colonies forming the peak with the highest intensity is about 0.44. This peak is deflected relative to the GD by negative 7.1 degrees. Five groups of dendrite colonies with a total volume fraction of about 0.45 are deflected antiparallel to the TrD at angles from 2.9 to 11.1 degrees.

It is assumed that the variety of angles of misorientation in the plane perpendicular to the direction of ST is due to the conditions of heat removal. In directions parallel and antiparallel to TrD, there is an increased contribution of the radiation component of the heat removal to the walls of the vacuum chamber of the installation because of the significantly smaller transverse dimensions of the formed product (about 11 mm) relative to the longitudinal dimensions (about 56 mm).

As was established in [19] and noted above, the slope of the preferential dendrites GD relative to the vertical direction BD by the curvature of the solidification front in the melt bath in combination with the unidirectional strategy of 3D printing. Whereas the slope of the preferential GD direction relative to the vertical BD is due to the reliably established (on several dozens of additive products) feature of heat removal from the melt bath into the cooled working table via the substrate. As can be seen in Figure 2a, the distances from the symmetry plane of the additive product to the substrate fixtures are a_1_ = 9.6 mm and a_2_ = 31.3 mm. Places of substrate fixation by fastening devices are viewed as bright areas of the substrate surface. In the process of forming an additive product, heat removal via heat conduction into the cooled worktable is realized through the substrate and fixing devices. Due to the fact that the distance a_2_ is more than three times the distance a_1_, the temperature gradient shifts towards the smaller heat dissipation distance. This, in turn, leads to asymmetry of the solidification front in the plane perpendicular to the ST.

### 3.2. Features of the Elemental Composition

It was noted above that in the present study, the formation of the additive product of ZhS32 superalloy was carried out on a substrate of rolled sheet of austenitic steel SS321. The presence of dissolved chemical elements of the substrate in the first few layers of the additive product material was expected. Earlier work [19] showed, using ZhS6U superalloy as an example, that in the additive product material, at a distance of more than 8 mm from a similar substrate, the main element of the substrate (iron) and its inherent alloying elements are not detected. In order to identify the influence of the substrate in terms of dissolved elements in the material of the additive product, a detailed elemental analysis on metallographic cross sections throughout the height of the product was carried out. Elemental analysis was carried out in the process of SEM research by means of energy dispersive analysis. Obtained results of such analysis are shown in Table 3. In this table, underestimated content of analyzed elements is depicted in red and overestimated content of analyzed elements is depicted in blue relative to the grade composition of superalloy ZhS32. The presented data indicate that at distances of 8.0 mm and more, the main element of the substrate in the form of iron is not detected.

In this aspect, the result was similar to the study [19]. However, in the grade composition of the ZhS6U superalloy, the permissible iron content is twice as high as in the material of the present study.

It should be noted that the underestimated aluminum content in the additive product material practically along its entire height. Taking into account the results of the study [11], it can be established that aluminum evaporation directly correlates with the value of heat input in the process of additive product formation. It is possible to eliminate excessive aluminum evaporation in the process of additive product formation by an additional reduction in the amount of heat input or by compensating the aluminum content in the raw material. The first approach has been implemented in a number of experiments that are beyond the scope of this study.

### 3.3. Features of the Fine Structure

**Figure 7:**
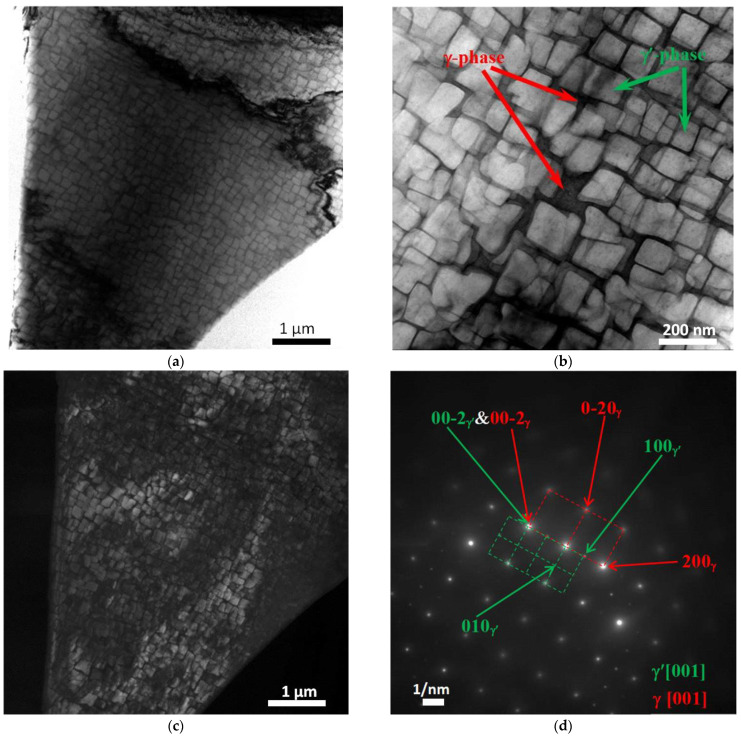
Fine structure of products material obtained by wire-feed electron beam additive manufacturing at a distance from the substrate of 40 mm (in the last layer). Bright-field (**a**,**b**) and dark-field (**c**) TEM images, SAED and its identification scheme (**d**). Dark-field image taken in the of reflection 100γ’.

**Figure 8:**
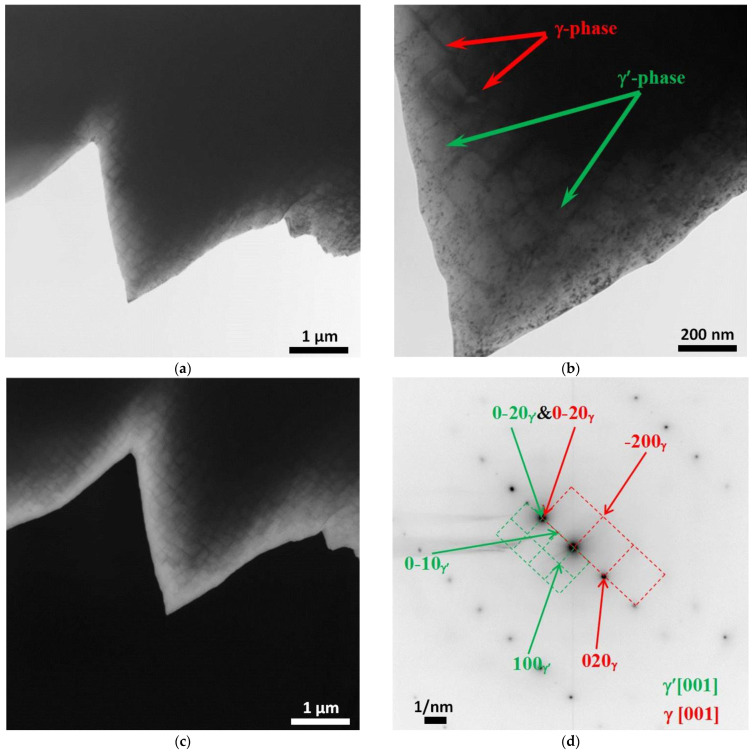
Fine structure of material of products obtained by wire-feed electron beam additive manufacturing at a distance from the substrate of 31 mm. Bright-field (**a**,**b**) and dark-field (**c**) TEM images, SAED and its identification scheme (**d**). Dark-field image taken in the reflection 0–10γ’.

**Figure 9:**
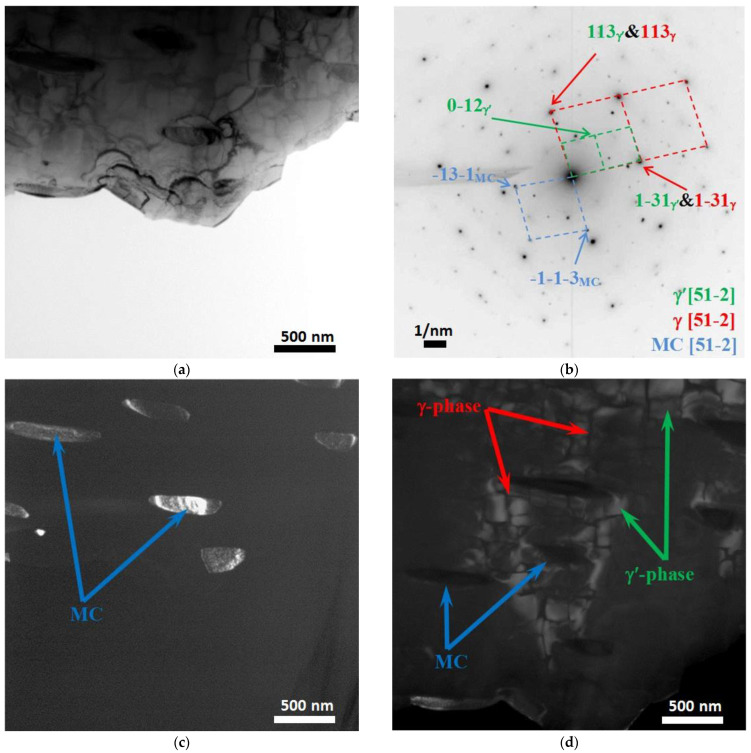
Fine structure of material of products obtained by wire-feed electron beam additive manufacturing at a distance from the substrate of 8 mm. Bright-field (**a**) and dark-field (**c**,**d**) TEM images, SAED and its identification scheme (**b**). Dark-field image taken in the reflection -13-1_MC_ (**c**) and 0–12_γ’_ (**d**).

**Figure 10:**
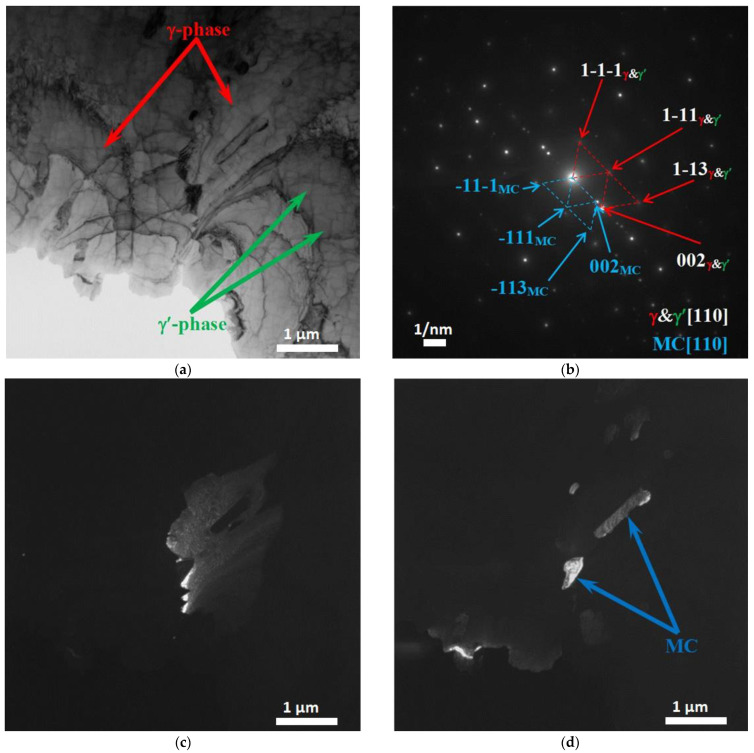
Fine structure of material of products obtained by wire-feed electron beam additive manufacturing at a distance from the substrate of 1 mm. Bright-field (**a**) and dark-field (**c**,**d**) TEM images, SAED and its identification scheme (**b**). Dark-field image taken in the reflection 002γ&γ’ (**c**) and 002MC (**d**).

**Figure 11:**
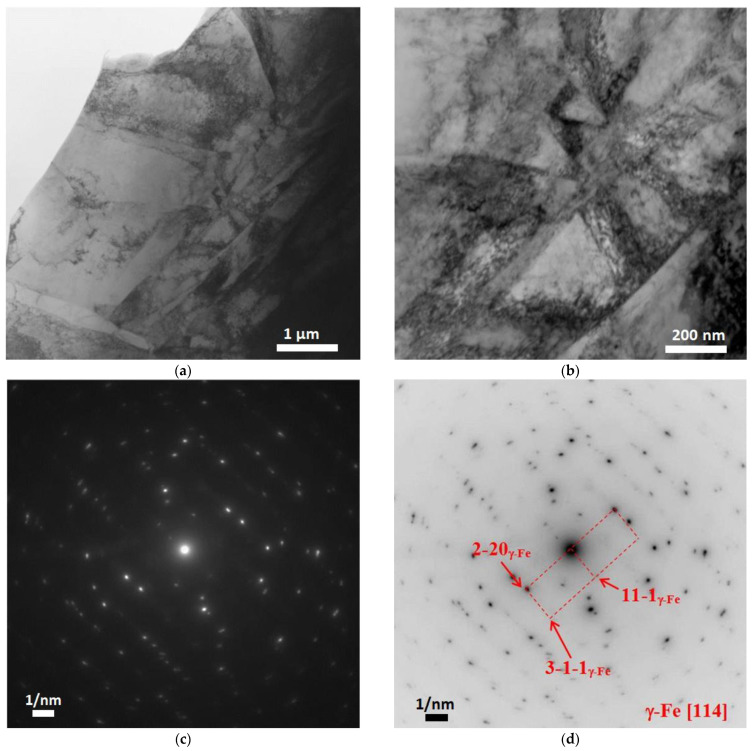
Fine structure of material of products obtained by wire-feed electron beam additive manufacturing right at the substrate (in the interlayer). Bright-field (**a**,**b**) TEM images, SAED (**c**) and its identification scheme (**d**).

Figure 7, Figure 8, Figure 9, Figure 10 and Figure 11 show images of the fine structure of the product material at distances of 40, 31, 8 and 1 mm from the substrate, as well as from the transition region directly at the substrate. At all distances from the substrate, except for the transition layer, the microstructure of the additive product material is the same. It consists of γ’-phase precipitations of the Al-Ni system (the phase based on the intermetallic compound AlNi_3_). The γ’-phase precipitates have cuboid morphology (predominantly cubes with smoothed edges). Between phase precipitates, there are interlayers of γ-phase (solid solution based on nickel). In addition to these phases, MC (where M: Nb + Ta + W) carbide precipitates were detected in the additive material (Figure 9 and Figure 10). Note that carbide precipitations are a characteristic feature of the microstructure and phase composition of additive products made of ZhS32 superalloy [23] throughout their height, except for the transition region near the substrate. Carbide precipitations are localized in the interdendritic space and have submicron sizes. As for the transition layer from the substrate to the additive product material, it formed an γ-Fe-based structure without γ’-phase separation of the Al-Ni system (Figure 11).

**Figure 12:**
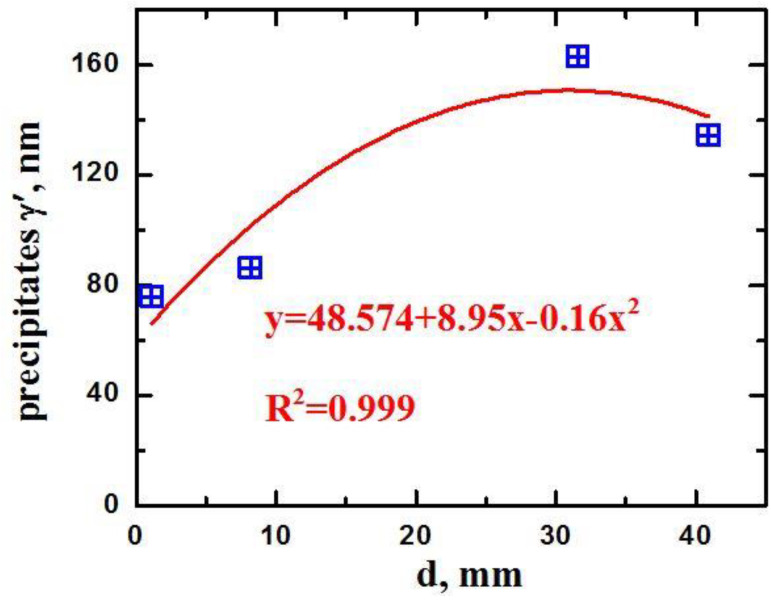
Dependence of the γ’-phase emission size on the distance from the substrate.

From the electron microscopic images using the secant method, the average dimensions of the γ’-phase excretions were determined. The results of measurements of γ’-phase cuboid sizes and subsequent mathematical processing of the measurement results showed the following (Figure 12). As the distance from the substrate increases, the average cuboid sizes increase. At the same time, the increase in the average cuboid sizes has a monotonic character with an extremum. At a distance of about 1 mm from the substrate, the average size of cuboids is 76 nm. At a distance of about 30 mm from the substrate, it reaches 163 nm. In the final layer of the product material, at a distance of about 41 mm from the substrate, the average size of cuboids did not exceed 135 nm. The extreme dependence of the size of γ’-phase precipitations along the height of the product is due to a combination of a given monotonic decrease in heat input and heat accumulation in the product material as it forms. In the process of 3D-printing of the final layer, in addition to heat removal through heat conduction through the previously formed layers of the product material and substrate into the cooled worktable, a significant contribution will be made by heat removal through radiation into the walls of the vacuum chamber of the installation.

## 4. Conclusions

Thus, the following features of macro-, micro-, and fine structure of the additive material of heat-resistant alloy ZhS32, formed by the method of electron beam additive technology, have been established:

- In the entire volume of the product material, there are no macro defects of discontinuities type (cracks, pores, voids, etc.);
- In the product material, at a distance of more than 8 mm from the substrate, no chemical elements of austenitic steel substrate material are revealed;
- The product material has a directed macrostructure, except for the final layer, not more than 3.5 mm thick. Directional macrostructure is represented by primary dendrite arms oriented predominantly along the {001} crystallographic direction. The mutual disorientation of dendrite axes does not exceed 9 degrees;
- The predominant growth direction of the primary dendrite axes is tilted by 23 degrees relative to the growth direction of the additive product in the direction of unidirectional 3D printing in the layers;
- The average primary dendrite arm spacing increases monotonically from 16 μm at a distance of 5 mm from the substrate to 23 μm in the final layers of the product material with a height of 41 mm;
- The average size of γ’-phase precipitations of the Ni-Al system in the form of nanoscale and submicrocrystalline cuboids, depending on the distance to the substrate, changes in the range from 76 to 163 nm. The size of γ’-phase precipitations reaches a maximum at a distance of about 30 mm from the substrate, and in the final layers of the product material, the average size of cuboids is 135 nm.

## Figures and Tables

**Table 1:** Chemical composition (% wt.) of material in the initial filament (in the form of bars) from nickel-based superalloy ZhS32.

Chemical Element	Al	Cr	Fe	Co	Nb	Mo	Ta	W	Re	Ni
Initial filament	5.73	4.99	0.11	9.27	1.57	1.16	4.03	8.48	3.88	Balance
Standard deviation	±0.1	±0.1	±0.1	±0.1	±0.1	±0.1	±0.3	±0.4	±0.3	

**Table 2:** Chemical composition (% wt.) of SS321 [16].

C	Mn	Si	P	S	Cr	Ni	N	Ti	Fe
0.08≤	2.00≤	0.75≤	0.045≤	0.030≤	17.0–19.0	9.0–12.0	0.1≤	5 × (C + N) ≤ 0.7	Balance

**Table 3:** Chemical composition (% wt.) of material additive component from nickel-based superalloy ZhS32. The colors show the underestimation of the brand composition of the regulated values in red and the overestimation in blue.

Chemical Element		Al	Cr	Fe	Co	Nb	Mo	Ta	W	Re	C
Superalloy ZhS32 [22]		5.7–6.2	4.5–5.3	≤0.5	9.0–9.5	1.4–1.8	0.9–1.3	3.7–4.4	8.1–8.9	3.6–4.3	0.13–0.20
Additive sample											
Distance from substrate, mm	3.0	5.42	5.52	5.10	8.85	1.56	1.16	4.20	9.08	3.84	not defined
	4.5	5.50	5.33	2.66	9.16	1.52	1.26	4.54	9.38	3.72	
	6.0	5.60	5.32	1.36	9.39	1.60	1.10	4.73	9.24	3.48	
	8.0	5.49	5.29	-	9.61	1.45	1.36	4.93	9.59	4.03	
	28.0	5.82	5.55	-	9.63	1.08	1.46	3.55	8.95	3.44	
	39.6	5.40	5.28	-	9.35	1.75	1.40	5.03	9.65	3.94	
	40.3	5.37	5.29	-	9.44	1.67	1.32	5.34	9.44	3.88	
Standard deviation		±0.1	±0.1	±0.1	±0.1	±0.1	±0.1	±0.3	±0.4	±0.3	-

## Data Availability

Data are contained within the article.
